# Molecular characterization of *Panstrongylus chinai* from northern Peru and its phylogenetic relationship to ecuadorian populations using the COI gene

**DOI:** 10.17843/rpmesp.2025.421.13976

**Published:** 2025-03-17

**Authors:** Archi Alejandro Ruiz-Polo, Angélica Maria Vigil-Correa, Lya Emilia Niño-Mendoza, Rosa Elena Santillan-Valdivia

**Affiliations:** 1 Entomology Research and Training Center, Luciano Castillo Colonna Subregional Health Directorate, Sullana, Piura, Peru. Entomology Research and Training Center Luciano Castillo Colonna Subregional Health Directorate Sullana Piura Peru; 2 Department of Biological Sciences, National University of Piura, Castilla, Piura, Peru. National University of Piura Department of Biological Sciences National University of Piura Castilla Piura Peru; 3 Vector Control Unit, Luciano Castillo Colonna Subregional Health Directorate, Sullana, Piura, Peru. Vector Control Unit Luciano Castillo Colonna Subregional Health Directorate Sullana Piura Peru

**Keywords:** Panstrongylus, phylogeny, DNA, bioinformatics

## Abstract

**Objective.:**

To determine the molecular characterization of Panstrongylus chinai from northern Peru and its phylogenetic relationship with Ecuadorian populations using the Cytochrome C Oxidase Subunit I (COI) gene.

**Materials and methods.:**

We analyzed three adult female P. chinai specimens from populations reared under laboratory conditions, from rural localities in the department of Piura. The legs of each specimen were dissected from the coxa to the tibia, discarding the tarsi and nails, then the DNA was extracted, and a polymerase chain reaction (PCR) of the COI gene was carried out. The PCR products were sequenced by Sanger and analyzed with DNA sequences of the COI gene of P. chinai from Ecuador, obtained from the NCBI portal, Genbank. The DNA sequences of the study, together with similar sequences found in the NCBI database, were inserted into the MEGA v.11 software to construct a phylogenetic tree. They were then transferred to the DnaSP v.5 software for molecular characterization by haplotypes.

**Results.:**

Molecular characterization revealed the presence of three haplotypes circulating in the department of Piura, different from the haplotype previously reported in Ecuador. Likewise, phylogenetic analysis suggests the emergence of the evolutionary process of cladogenesis, in which the Ecuadorian variant may have originated from populations of P. chinai from northern Peru.

**Conclusions.:**

P. chinai from Ecuador and northern Peru have different molecular characteristics and a descending phylogeny, which infer distribution from Peru to Ecuador.

## INTRODUCTION

Triatomines are insect vectors known for their role in transmitting the *Trypanosoma cruzi* parasite, which causes American trypanosomiasis, also known as Chagas disease ^1^. From 2000 to 2023, the number of Chagas cases in Peru reached a cumulative total of 1,270 confirmed cases, of which 63 were reported in 2023 ^2^. However, 118 confirmed cases were reported in Ecuador in the same year, ^3^.

Among the species of triatomines associated with this disease, 18 have been identified in Peru, distributed across seven genera ^4^, which are classified into three groups: domestic, which reside inside dwellings; peridomestic, which are found in domestic animal breeding sites; and wild, which inhabit nests and caves of wild animals ^5^.

Species such *Triatoma infestans* in Peru are distributed in the southern regions of the country ^6^. Around 17 species have been documented in northern Peru, with *Panstrongylus chinai* being the most prevalent ^7^, frequently found in the intertropical region, particularly in countries such as Ecuador, Peru, and Venezuela, as well as in other areas of South America ^8^. However, Ecuador and Peru are the countries with most frequent reports ^9^, which is attributed to climate adaptation processes that have favored potential speciation and endemism in certain geographical areas ^10^^-^^12^.

In this context, classical taxonomy can be used to identify these insects, which is based on the evaluation and recognition of morphological characteristics, such as sexual dimorphism, wing size, and head structure, which are used as phenotypic markers to differentiate between wild and domestic populations ^13^^-^^16^. Alternatively, molecular taxonomy can be used to find genetic changes and analyze the geographical distribution of lineages at the intraspecific level, using DNA sequences from mitochondrial markers, which can be used to characterize and differentiate several populations of species through haplotype typing ^17^^,^^18^.

The Cytochrome C Oxidase Subunit I (COI) is one of the most commonly used markers ^19^. The selection of this and other mitochondrial markers is justified by their small size, conserved genetic organization, and high mutation rate, estimated at 5.7 x 10⁻⁸ per site per year. In addition, universal primers are used for amplification ^20^^,^^21^. These properties make mitochondrial DNA relatively accessible for analysis and show remarkably high levels of polymorphism, which often indicates the presence of multiple genetic lineages both within and between populations ^21^.

The rapid and accurate identification of species in a given transmission area is highly important for public health ^22^. Therefore, phylogenetic studies are essential for detecting cladogenesis events, as they allow the evaluation of the degree of nucleotide divergence and the mutation rate characteristic of each taxon ^23^.

In light of the above, it is essential to conduct molecular studies that assess the distribution of triatomines in South America, as the obtained data are crucial for formulating preventive strategies aimed at vector control in regions bordering neighboring countries that are endemic for Chagas disease. This type of research facilitates the identification of potential routes of infection and analyze mutations that could indicate an increase in transmission capacity. In this study, we determined the molecular characterization of *P. chinai* from northern Peru and its phylogenetic relationship with Ecuadorian populations using the COI gene.

**KEY MESSAGES**

Motivation for the study. Studies on the molecular characterization and phylogenetic analysis of *Panstrongylus chinai* in South America are limited, despite their significant contribution to the biological understanding of the vector and its genetic variations, which allow hypothesizing about its origin, dispersion, and phylogenetic evolution.

Main findings. It is proposed that lineages of *P. chinai* from northern Peru gave rise to lineages in Ecuador as a result of migration between the two countries.

Implications. These findings can be used as theoretical basis for new studies of *P. chinai* between Peru and Ecuador, or South America in general, helping to clarify the routes of dispersal on the continent and, as a result, the emergence of cases of Chagas disease.

## MATERIALS AND METHODS

### Study design

A descriptive, non-experimental *in vitro* study was conducted in the molecular biotechnology laboratory of the Entomology Research and Training Center (CICE) of the Luciano Castillo Colonna Subregional Health Directorate (DSRSLCC), located in the district of Querecotillo, province of Sullana, in the department of Piura, Peru (supplementary material). The study focused on the molecular characterization of *P. chinai* from northern Peru and its phylogenetic relationship with Ecuadorian populations using the COI gene. Therefore, genetic material from live specimens collected in three rural locations in Peru was used to obtain sequences of the COI gene, which were then compared with those of *P. chinai* from Ecuador available in the gene bank.

### Samples and bioinformatic material

The samples correspond to three adult female specimens of *P. chinai*, each from different populations: Jibito (4°54′16″S/80°44′47″W), Tambogrande (4°55'36.98“S/80°20'40.99”W) and Las Lomas (4°39'11.99“S/80°14'48.01”W). These specimens were provided by the CICE insectarium, which has bred generations of these insects in controlled laboratory conditions.

The bioinformatic material corresponds to sequences of the COI gene from populations of *P. chinai* from Ecuador available in the gene bank (Genbank) of the National Center for Biotechnology Information (NCBI) https://www.ncbi.nlm.nih.gov/. The sequences from Genbank were deposited by Barnabé *et al*. ^24^ with the following access codes: MN504941 and MN504934.

### DNA extraction

The specimens were sacrificed by exposing them to -20 °C for 20 minutes. After this time, the legs were dissected from the coxa to the tibia, discarding the tarsus and claws using a sterile scalpel, and then washed in ultrapure water (UPW). The muscle tissue was then extracted and distributed into sterile 1.5 mL microtubes, 300 µL of 0.9% sodium chloride (NaCl) was added to each microtube, left to stand for 5 minutes at room temperature, and crushed by maceration with sterile polypropylene pestles. Finally, DNA was extracted using the Zymo Biomics DNA Miniprep Kit (Cat. D4300), following the manufacturer’s instructions and modifying the cell lysis step, omitting the use of ZR Bashing Bead™ Lysis Tubes and using a pestle instead.

### PCR targeting the COI gene

After obtaining total DNA from the specimens, the COI gene was amplified by polymerase chain reaction (PCR), expecting a 710 bp amplicon according to the primers described by Folmer *et al*. *(25)* (LCO1490Forward: '5-GGTCAACAAATCATAAAGATATTGG-3'/HCO2198Reverse: '5-TAAACTTCAGGGTGACCAAAAAATCA-3'). The reaction was performed using the GoTaq™ G2 Flexi DNA Polymerase PCR kit (Promega M7801), according to the manufacturer’s instructions. The final volume was 50 µL, containing 22.5 µL of nuclease-free water, 10 µL of buffer (1X), 3 µL of MgCL (1.5 mM), 1 µL of dNTPs (200 µM), 2.5 µL of Forward LCO1490 (10 uM), 2.5 µL of Reverse HCO2198 (10 uM), 0.5 µL of Gotaq Polymerase enzyme (1U/reaction), and 8 µL of total DNA. The thermal conditions and cycling consisted of an initial denaturation at 95 °C for 5 minutes, followed by 35 cycles at 95 °C for 30 seconds for denaturation, 58 °C for 30 seconds for hybridization, 72 °C for 1 minute for extension, a post-extension of 72 °C for 5 minutes, and a storage temperature of 4 °C for up to 24 hours.

The obtained PCR products (amplicons) were visualized by agarose gel electrophoresis in 2% TAE (Tris-Acetate-EDTA) solution stained with 4.5 µL of ethidium bromide and 8 µL of sample (extracted DNA). For DNA migration, 4 µL of loading buffer (6X DNA loading dye) and 2 µL of 1000 bp (1kb) molecular weight marker (Opti-DNA Marker G016) were used. This validated the amplification of the COI gene DNA sequence.

### DNA sequencing

After validating the amplification of the COI gene, these were sent to the biotechnology company Macrogen (https://dna.macrogen.com/) for DNA sequencing using double-strand Sanger technology. The sequences were then delivered in FASTA format (a text-based computer file format) for molecular characterization and phylogeny analysis.

### Molecular characterization and phylogeny

The generated COI gene DNA sequences were released in FASTA format and analyzed using the bioinformatics program Molecular Evolutionary Genetics Analysis version 11 (MEGA 11, https://mega.io/es), which generated consensus sequences from the direct and reverse readings of each sample. They were then inserted into the basic local alignment search tool (BLAST, https://blast.ncbi.nlm.nih.gov/Blast.cgi), where they were searched for similarities with sequences from Ecuador available on the Genbank portal.

For molecular characterization, similar sequences were aligned with those in this study, then processed in the DnaSP v.5 program. The option “Open Unphase/Genotype Data file” was selected for import because these were sequences from a diploid organism. The polymorphisms, their location, and the haplotypes they gave rise to were immediately obtained using the “Generate Haplotype Data File” option in a text file.

Finally, for the evolutionary analysis, we constructed a phylogenetic tree in MEGA11 using the neighbor joining method ^26^, under Tamura's 3-parameter model ^27^^)^ with 500 bootstraps, in addition to a sequence from *P. rufotuberculatus* as an outgroup (MZ643673).

### Data analysis

The haplotypes were organized in Microsoft Excel Version 2021 spreadsheets, indicating the polymorphisms included in each one.

### Ethical considerations

In this study we used live specimens of *P. chinai* from the CICE, so it was not necessary to request specific permits for their analysis. However, for the use of genetic information corresponding to the COI gene of *P. chinai* from Ecuador, which is available in the NCBI genetic database, the corresponding access code (MN504941 and MN504934) was included, and the authors of this information were cited and referenced.

## RESULTS

### Electrophoresis of PCR products

**Figure 1:**
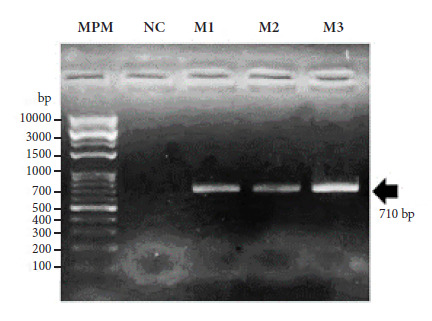
Agarose gel electrophoresis of PCR products directed at the COI gene. MPM: 1KB molecular weight marker. NC: Negative control. M1: *Panstrongylus chinai* from Jibito. M2: *Panstrongylus chinai* from Tambogrande. M3: *Panstrongylus chinai* from Las Lomas.

PCR product aimed at amplifying the COI gene of the analyzed *P. chinai* specimens yielded amplicons with a molecular weight of 710 base pairs (Figure 1).

bp: base pairs

### Molecular characterization

As a result of sequencing, we obtained COI gene sequences of 650 base pairs (bp), which were aligned to generate consensus sequences of the same size. After removing four sites with gaps, the sequences were reduced to a final consensus of 646 bp. The final consensus sequences were compared with those deposited in the genetic database for Ecuador (access codes MN504941 and MN504934), showing a high similarity in a region of 393 bp, which was used for the molecular characterization of *P. chinai*.

Of the 393 bp analyzed DNA regions, we excluded one site with a gap, resulting in a final region of 392 bp. In this segment, 387 monomorphic sites and 5 polymorphic sites were identified, located at genetic positions 12, 189, 195, 226, and 252. Six mutations were found, corresponding to the nucleotides highlighted in yellow (G, A, T), which led to the formation of 4 haplotypes: Hap-1, Hap-2, Hap-3, and Hap-4. These haplotypes were distributed into 3 groups, one for each location in northern Peru, while a single haplotype was identified for Ecuador (Table 1).

**Table 1:** Molecular characteristics of the COI gene DNA sequences analyzed.

Species	Origin		Sequence source	Haplotypes	Genetic position				
	Country	Code			12	189	195	226	252
*P. chinai*	Ecuador	MN504941	Genbank	Hap-1	A	T	G	C	C
*P. chinai*	Ecuador	MN504934	Genbank		A	T	G	C	C
*P. chinai*	Peru	M1	Own	Hap-2	A	T	A	C	C
*P. chinai*	Peru	M2	Own	Hap-3	G	A	A	T	A
*P. chinai*	Peru	M3	Own	Hap-4	A	T	A	C	T

### Phylogenetic analysis

**Figure 2:**
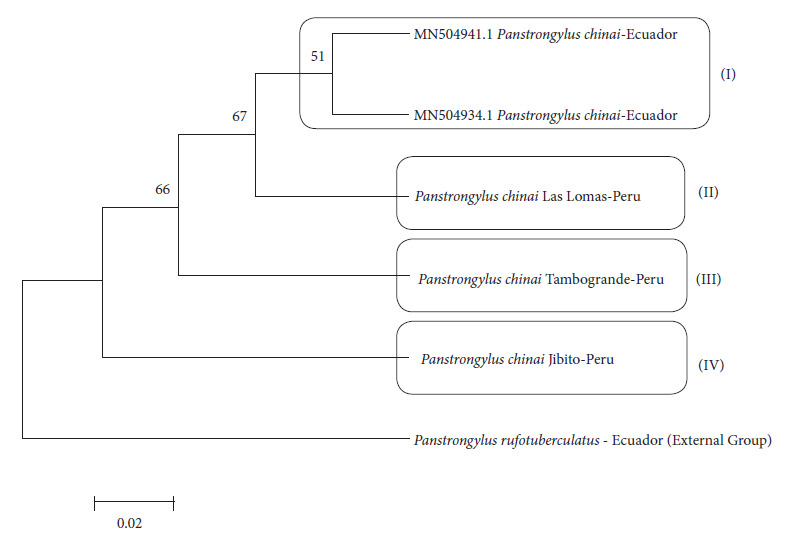
Phylogenetic tree based on the *Neighbor Joining* distance method ^24^^)^ and Tamura's 3-parameter model ^25^^)^ with a *bootstrap* value of 500 replicates, showing the relationship between the haplotypes of the mitochondrial COI gene found in the DNA sequences of the specimens from northern Peru in this study and the DNA sequences from Ecuador obtained from *Genbank*.

The phylogenetic tree, generated using the Neighbor Joining method, allowed us to infer the evolutionary relationships between the identified haplotypes during molecular characterization. This analysis supports an evolutionary hypothesis in which *P. chinai* from Ecuador forms a monophyletic clade in relation to specimens from northern Peru. The haplotype of *P. chinai* from Ecuador (Hap-1) is more recent than the haplotypes from Peru (Hap-2, Hap-3, and Hap-4), suggesting that the Ecuadorian variant may have arisen from Peruvian populations of *P. chinai* (Figure 2).

Four clades can be found in the phylogenetic tree: one exclusive to Ecuador (I) and three corresponding to Peru (II, III, IV). Clades I, II, and III are located on subsequent branches that emerge from a branch shared with clade IV, indicating that they all originate from the same node, suggesting a common ancestor. This pattern is interpreted as a cladogenesis event, where an ancestral lineage has given rise to sister lineages.

## DISCUSSION

Triatomine species exhibit a high degree of morphological plasticity and variability in their geographical distribution ^28^. For this reason, entomologists have been conducting a comprehensive analysis of several groups of triatomines that exhibit morphological differences, which are often subtle ^29^. This highlights the importance of conducting molecular studies on these insects, as it is possible to unequivocally clarify the existence of evolutionary events, such as speciation between populations of the same species.

In this study, DNA fragments of the COI gene were amplified from 710 bp from specimens of *P. chinai* from Peru, of which a 392 bp region was used for molecular characterization. After this analysis we identified four haplotypes (Hap-1, Hap-2, Hap-3, and Hap-4), distributed as one haplotype for Ecuador and three haplotypes for Peru, demonstrating remarkable genetic variability among populations of this triatomine species. This finding is consistent with previous studies that have documented genetic variations in geographically distant populations of *Triatoma infestans*, which reported divergence at the species level ^30^, as well as in other species of the genus *Triatoma*^31^. These variations suggest that geography plays a fundamental role in the genetic differentiation of populations. Similarly, studies conducted with *P. herreri* have found molecular differences within the same population, which was interpreted as possible speciation in progress ^32^. This phenomenon could explain the genetic divergence observed in *P. chinai* in our study, since the populations in Peru and Ecuador are geographically separated, which could have favored the accumulation of independent mutations in each population. Likewise, the mutation rate of the COI gene per site per year ^21^ facilitated the identification of polymorphisms that may be indicative of evolutionary processes and divergence between populations. The mitochondrial marker COI has been widely used in biodiversity and phylogeography studies, especially in invertebrate organisms such as insects ^33^. Previous studies on the genus *Panstrongylus* used the mitochondrial COI marker due to its high mutation rate and ability to reflect genetic variations within populations, allowing differences resulting from local evolutionary processes or geographical isolation to be inferred ^34^. However, in recent decades, other molecular markers such as ribosomal DNA ITS-2 have also been used in this type of study, as they have proven effective in differentiating between populations and species of triatomines ^35^^,^^36^. Similarly, Jerí and Solis ^37^ used this marker for the genetic differentiation of triatomine species in different geographical areas.

Therefore, from a theoretical point of view, the existence of genetic polymorphisms among populations of the same species is due to the accumulation of mutations in genomes over successive generations, resulting in the formation of distinct haplotypes ^38^, which can be observed in different molecular markers such as COI and ITS-2, reinforcing the existence of genetic variations between geographically separated triatomines. Therefore, in our study, the differences in haplotypes found between *P. chinai* populations in Peru and Ecuador can be explained by this mechanism, which reflects genetic differentiation driven by geographical separation and the accumulation of mutations over time.

The results from the phylogenetic analysis based on COI gene sequences suggest a process of cladogenesis in *P. chinai*, pointing to a descending origin of the Ecuadorian populations from populations in the department of Piura, located in northern Peru. This pattern of phylogenetic relationships is consistent with the geographical and ecological proximity of both regions. The populations of *P. chinai* in the provinces of Loja and El Oro, located in the southern Andean region of Ecuador, are adjacent to northern Peru ^39^, providing a favorable biogeographic context for the exchange of individuals and genetic material between populations in both countries. The geographical proximity, together with the fact that the DNA sequences from Ecuador analyzed in this study come from specimens captured in 2019 in areas close to the border with Peru ^24^, reinforces the hypothesis of a historical and continuous link between these populations. The movement of *P. chinai* from Peru to Ecuador and its relationship in terms of geographical distribution does not appear to be a recent phenomenon. In fact, a comprehensive study conducted in 2010 in southern Ecuador, specifically in rural areas of the province of Loja, documented the presence of *P. chinai* in both indoor and peridomestic environments. In addition, other triatomine species were found in that region, such as *R. ecuadoriensis*, *T. carrioni*, and *P. rufotuberculatus*^40^. Reports on species of the genus *Panstrongylus* in Peru ^41^^)^ indicate that *P. chinai* is one of the most prevalent species in the northern and northeastern regions of the country ^42^, highlighting its importance as a vector of *Trypanosoma cruzi*, the etiological agent of Chagas disease. The predominance of this species in specific areas of Peru is relevant for understanding the transmission patterns of the parasite and the dynamics of the disease. In this regard, the distribution of *P. chinai* in the country has been documented in several studies, which indicate its presence since at least 1961 ^43^, with new reports in 1986 ^44^, suggesting a stable and prolonged presence in these geographical areas. Therefore, our results can be interpreted, as they show a phylogenetic connection between the populations of *P. chinai* in Peru and Ecuador, which is based on the existence of continuous genetic flow and exchange between the populations of both countries. The fact that *P. chinai* populations in the border regions of Peru and Ecuador share similar ecological characteristics facilitates the spread of the species and, potentially, of the *T. cruzi* parasite. In addition, the geographical proximity between the endemic regions of both countries favors constant interaction between triatomine populations, which increases the likelihood that this flow will continue.

The public health implications of our study are closely related to the spatial distribution of *P. chinai* in northern Peru and Ecuador, as both countries are located in a region of South America where cases of Chagas disease have been reported for several decades. This finding is particularly relevant in the context of increasing urbanization, migration from rural areas, and the effects of climate change, all of which are altering the epidemiological patterns of the disease. According to the World Health Organization (WHO), climate change and migratory movements have transformed the epidemiological landscape of Chagas disease, contributing to its spread and making it a global condition in a relatively short time ^45^. In Peru, 57 confirmed cases and 7 probable cases of Chagas disease were reported in 2023, with an update for epidemiological week 11 of 2024 reflecting 3 confirmed cases and 9 probable cases ^47^. In Ecuador, 118 confirmed cases were reported during 2023 ^3^. These data underscore the importance of continuing epidemiological surveillance and implementing control strategies, especially in geographic areas where the distribution of *P. chinai* could contribute to the maintenance of transmission cycles.

Our study had some limitations that should be considered to enrich and improve the interpretation of the results. One of the main limitations was the limited bibliographic information available on the subject of study, which makes it difficult to compare the findings with previous research. In addition, the amount of genetic data on the COI gene of *P. chinai* in the gene bank is limited. In this regard, we found only sequences deposited by Barnabé *et al*. ^24^, which limits the possibility of conducting a more comprehensive comparative analysis with other populations or related species. In fact, this study represents one of the few studies in which the COI gene has been analyzed in *P. chinai*, highlighting the importance of generating and sharing more genetic data on this species for future research.

In conclusion, our study provides an overview of the spatial distribution of *P. chinai* between northern Peru and Ecuador, analyzing its genetic variations. We identified molecular characteristics that distinguish populations in terms of polymorphisms, although phylogenetically they are related in terms of cladogenesis and common ancestry. In this regard, an evolutionary hypothesis is proposed, in which the populations of *P. chinai* in northern Peru gave rise to the populations present in Ecuador. These results highlight the importance of conducting additional molecular studies on the distribution of triatomines in South America, as the information obtained will contribute to the design of preventive strategies for vector control, especially in areas bordering countries where Chagas disease is endemic.
