# Supplementary material for: Molecular characterization of Panstrongylus chinai from northern Peru and its phylogenetic relationship to ecuadorian populations using the COI gene
Source: Rev Peru Med Exp Salud Publica. 2025 Mar 17;42(1):6–13. doi: 10.17843/rpmesp.2025.421.13976 (PMC12176026; doi:10.17843/rpmesp.2025.421.13976)
Supplement: Supplementary material. — Available in the electronic version of the RPMESP. [file rpmesp-42-01-13976-s001.docx]

**Material suplementario**


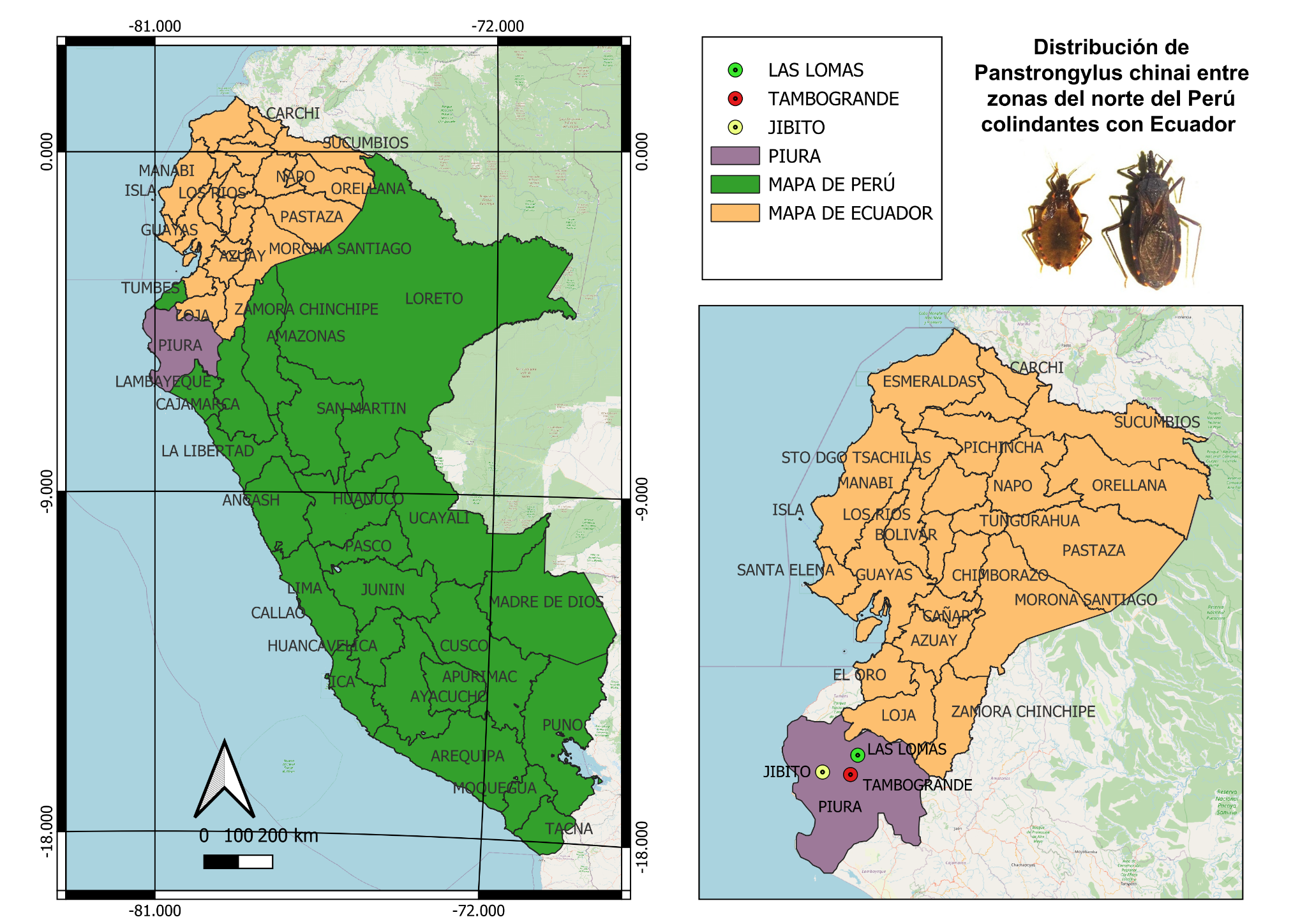


**Figura 1S.** Ubicación geográfica de las localidades del norte del Perú colindantes con Ecuador en donde se han capturado los especímenes *P. chinai* analizados. El mapa color verde representa a Perú. El mapa color morado representa al departamento de Piura (Norte del Perú). Los puntos amarillo, rojo y verde en el mapa color morado representan las localidades de captura. El mapa color naranja representa a Ecuador.
