# Supplementary material for: Molecular characterization of Panstrongylus chinai from northern Peru and its phylogenetic relationship to ecuadorian populations using the COI gene
Source: Rev Peru Med Exp Salud Publica. 2025 Mar 17;42(1):6–13. doi: 10.17843/rpmesp.2025.421.13976 (PMC12176026; doi:10.17843/rpmesp.2025.421.13976)
Supplement: Translation

# Translation: Caracterización molecular de *Panstrongylus chinai* del norte del Perú y su relación filogenética con poblaciones ecuatorianas empleando el gen COI

**Authors:** Archi Alejandro Ruiz-Polo, Angélica Maria Vigil-Correa, Lya Emilia Niño-Mendoza, Rosa Elena Santillan-Valdivia

**Affiliations:** 1 Centro de Investigación y Capacitación en Entomología, Dirección Sub Regional de Salud Luciano Castillo Colonna, Sullana, Piura, Perú.; 2 Departamento de Ciencias Biológicas, Universidad Nacional de Piura, Castilla, Piura, Perú.; 3 Unidad de Control Vectorial, Dirección Sub Regional de Salud Luciano Castillo Colonna, Sullana, Piura, Perú.

**Keywords:** Panstrongylus, filogenia, ADN, bioinformática

## Abstract

**Objetivo.:**

Determinar la caracterización molecular de *Panstrongylus chinai* del norte del Perú y su relación filogenética con poblaciones ecuatorianas empleando el gen *Citocromo C Oxidasa Subunidad I* (COI).

**Materiales y métodos.:**

Se analizaron tres ejemplares adultos hembras de *P. chinai* de poblaciones criadas en condiciones de laboratorio, que corresponden a localidades rurales del departamento de Piura. En cada ejemplar se disectaron las patas desde la coxa hasta la tibia descartando los tarsos y uñas, se extrajo ADN, y se llevó a cabo una reacción en cadena de la polimerasa (PCR) del gen COI. Los productos PCR se secuenciaron por Sanger y se analizaron con secuencias ADN del gen COI de *P. chinai* de Ecuador, obtenidas del portal de NCBI, Genbank. Las secuencias ADN del estudio con sus similares halladas en la base de datos del NCBI, se insertaron en el software MEGA v.11 para la construcción de un arbol filogenético. Luego, se transfirieron al software DnaSP v.5 para su caracterización molecular mediante haplotipos.

**Resultados.:**

La caracterización molecular reveló la presencia de tres haplotipos circulantes en el departamento de Piura, diferentes al haplotipo previamente reportado en Ecuador. Así mismo, el análisis filogenético sugiere la aparición del proceso evolutivo de cladogénesis, en el cual la variante ecuatoriana pudo haberse originado a partir de poblaciones de *P. chinai* del norte del Perú.

**Conclusiones.:**

*P. china*i de Ecuador y del norte del Perú presentan características moleculares distintas y una filogenética descendente, que infieren una distribución de Perú hacia Ecuador.

## INTRODUCCIÓN

Los triatominos son insectos vectores reconocidos por su papel en la transmisión del parásito *Trypanosoma cruzi*, responsable de la tripanosomiasis americana, también conocida como enfermedad de Chagas ^1^. Desde el año 2000 al 2023, el número de casos de Chagas en el Perú alcanzó una cifra acumulada de 1270 casos confirmados, de los cuales 63 se han reportado en el año 2023 ^2^. No obstante, para el mismo año en Ecuador se reportaron 118 casos confirmados ^3^.

Entre las especies de triatominos asociadas a esta enfermedad, en Perú se han identificado 18, hallándose distribuidas en siete géneros ^4^, que se clasifican en tres grupos: los domésticos que residen en el interior de las viviendas; los peridomésticos que se localizan en criaderos de animales domésticos y los silvestres, que habitan en nidos y cuevas de fauna silvestre ^5^.

 En lo que respecta a su distribución geográfica en el Perú, al sur se ha registrado la presencia de especies como *Triatoma infestans*^6^. En el norte, se han documentado alrededor de 17 especies, siendo *Panstrongylus chinai* la más prevalente ^7^, encontrándose frecuentemente en la región intertropical, especialmente en países como Ecuador, Perú y Venezuela, así como en otras áreas de Sudamérica ^8^. No obstante, Ecuador y Perú son los países donde se ha reportado con mayor frecuencia ^9^, lo cual se atribuye a procesos de adaptación climática que han favorecido la potencial especiación y endemismo en ciertas zonas geográficas ^10^^-^^12^.

En ese contexto, una de las metodologías para identificar estos insectos es la taxonomía clásica que se fundamenta en la evaluación y el reconocimiento de caracteres morfológicos, tales como el dimorfismo sexual, el tamaño de las alas y la estructura de las cabezas, los cuales se utilizan como marcadores fenotípicos para diferenciar entre poblaciones silvestres y domiciliarias ^13^^-^^16^. Alternativamente, se puede emplear la taxonomía molecular, que permite observar cambios genéticos y analizar la distribución geográfica de linajes a nivel intraespecífico, utilizando secuencias de ADN de marcadores mitocondriales, con los que se puede realizar la caracterización y diferenciación molecular de diversas poblaciones de especies mediante tipificación haplotípica ^17^^,^^18^.

Entre los marcadores más utilizados se encuentra la Subunidad I del Citocromo C Oxidasa (COI) ^19^. La selección de este y otros marcadores mitocondriales se justifica por su tamaño reducido, su organización genética conservada y su alta tasa de mutación, estimada en 5,7 x 10⁻⁸ por sitio por año. Además, se emplean cebadores universales para su amplificación ^20^^,^^21^. Estas propiedades hacen que el ADN mitocondrial sea relativamente accesible para el análisis y que muestre niveles de polimorfismo notablemente elevados, lo que frecuentemente indica la presencia de múltiples linajes genéticos tanto dentro de las poblaciones como entre ellas ^21^.

La identificación rápida y precisa de las especies presentes en una determinada área de transmisión es de suma importancia para la salud pública ^22^. Por lo tanto, los estudios filogenéticos son esenciales para detectar eventos de cladogénesis, ya que permiten evaluar el grado de divergencia nucleotídica y la tasa de mutación características de cada taxón ^23^.

En virtud de lo mencionado anteriormente, es esencial llevar a cabo estudios moleculares que evalúen la distribución de triatominos en Sudamérica, puesto que los datos obtenidos son cruciales para formular estrategias preventivas orientadas al control vectorial en regiones limítrofes con países vecinos que son endémicos para la enfermedad de Chagas. A través de este tipo de investigaciones, es posible identificar potenciales rutas de infección y analizar mutaciones que podrían indicar un aumento en la capacidad de transmisión. En el presente estudio se determinó la caracterización molecular de *P. chinai* del norte del Perú y su relación filogenética con poblaciones ecuatorianas empleando el gen COI.

**MENSAJE CLAVE**

Motivación para el estudio. Los estudios sobre caracterización molecular y análisis filogenético de *Panstrongylus chinai* en Sudamérica son limitados, a pesar de su significativa contribución a la comprensión biológica del vector y sus variaciones genéticas, que permiten formular hipótesis sobre su origen, dispersión y evolución filogenética.

Principales hallazgos. Se propone que linajes de *P. chinai* procedentes del norte del Perú habrían dado origen a linajes en Ecuador, como resultado del desplazamiento entre ambos países.

Implicaciones. Estos hallazgos podrán ser usados como base teórica para nuevos estudios de *P. chinai* entre Perú y Ecuador, o Sudamérica en general, permitiendo esclarecer las rutas de dispersión en el continente y como consecuencia, la aparición de casos de Chagas.

## MATERIALES Y MÉTODOS

### Diseño del estudio

Se llevó a cabo un estudio *in vitro* de diseño no experimental, de tipo descriptivo, en el laboratorio de biotecnología molecular del Centro de Investigación y Capacitación en Entomología - (CICE) de la Dirección Subregional de Salud Luciano Castillo Colonna (DSRSLCC), ubicado en el distrito de Querecotillo, provincia de Sullana, en el departamento de Piura, Perú (material suplementario). El estudio se centró en la caracterización molecular de *P. chinai* del norte de Perú y su relación filogenética con poblaciones ecuatorianas mediante el gen COI. Para ello, se utilizó material genético de ejemplares vivos recolectados en tres localidades rurales de Perú, a partir de las cuales se obtuvieron las secuencias del gen mencionado, las cuales se compararon posteriormente con las de *P. chinai* de Ecuador disponibles en el banco de genes.

### Muestras y material bioinformático

Las muestras analizadas corresponden a tres ejemplares adultos hembras de *P. chinai*, cada uno procedente de diferentes poblaciones: Jibito (4°54′16″S/80°44′47″O), Tambogrande (4°55’36.98”S/80°20’40.99”O) y Las Lomas (4°39’11.99”S/80°14’48.01”O). Estos ejemplares fueron proporcionados por el insectario del CICE, donde forman parte de generaciones criadas en condiciones controladas de laboratorio.

En cuanto al material bioinformático analizado, este corresponde a secuencias del gen COI de poblaciones de *P. chinai* de Ecuador disponibles en el banco de genes (Genbank) del Centro Nacional para la Información Biotecnológica (NCBI, por sus siglas en inglés) https://www.ncbi.nlm.nih.gov/. Las secuencias analizadas del Genbank han sido depositadas por Barnabé *et al*. ^24^ con los siguientes códigos de acceso: MN504941 y MN504934.

### Extracción de ADN

Los ejemplares fueron sacrificados al exponerse a -20 °C durante 20 minutos. Transcurrido el tiempo, se disectaron las patas desde la coxa hasta la tibia descartando el tarso y las uñas mediante un bisturí estéril, luego se lavaron en agua ultrapura (AUP). Posteriormente, se extrajo el tejido muscular y se distribuyó en microtubos estériles de 1,5 mL, se añadieron 300 uL de cloruro de sodio (NaCl) al 0,9% en cada microtubo, se dejó reposar por 5 minutos a temperatura ambiente y se trituró por macerado con pistilos de polipropileno estériles. Finalmente, se extrajo el ADN empleando el Kit Miniprep Zymo Biomics DNA (Cat. D4300), siguiendo las instrucciones del fabricante y modificando el paso de lisis celular, omitiendo el uso de los tubos ZR Bashing Bead™ Lysis Tubes por un macerado con pistilos.

### PCR dirigida al gen COI

Luego de obtener el ADN total de los ejemplares, se realizó la amplificación del gen *COI* mediante reacción en cadena de la polimerasa (PCR, por sus siglas en inglés) esperando un amplicón de 710 pb de acuerdo con los cebadores descritos por Folmer *et al.*^25^ (LCO1490Forward:‘5-GGTCAACAAATCATAAAGATATTGG-3’/HCO2198Reverse:‘5-TAAACTTCAGGGTGACCAAAAAATCA-3’). La reacción se realizó utilizando el kit PCR GoTaq™ G2 Flexi DNA Polymerase (Promega M7801), de acuerdo con las indicaciones del fabricante. El volumen final de la mezcla fue de 50 µL, conteniendo 22,5 µL de agua libre de nucleasas, 10 µL de buffer (1X), 3 µL de MgCL (1,5 mM), 1 µL de dNTPs (200 µM), 2,5 µL de Forward LCO1490 (10 uM), 2,5 µL de Reverse HCO2198 (10 uM), 0,5 µL de enzima Gotaq Polimerasa (1U/reacción) y 8 µL del ADN total. Las condiciones térmicas y el ciclaje consistieron en una desnaturalización inicial de 95 °C por 5 minutos, seguido de 35 ciclos con 95 ºC por 30 segundos para la desnaturalización, 58 °C por 30 segundos para la hibridación, 72°C por 1 minuto para la extensión, una posextensión de 72 °C por 5 minutos y una temperatura de conservación de 4 °C por hasta 24 horas.

Los productos PCR (amplicones) obtenidos se visualizaron mediante electroforesis en gel de agarosa al 2% en solución TAE 1X (Tris-Acetato-EDTA) teñidos con 4,5 uL de Bromuro de Etidio y 8 µL de muestra (ADN extraído). Para la migración del ADN se usó 4 uL de buffer de carga (6X DNA loading dye) y 2 uL de marcador de peso molecular de 1000 pb (1kb) (Opti-DNA Marker G016). Esto validó la amplificación de la secuencia ADN del gen COI.

### Secuenciación de ADN

Luego de validar la amplificación del gen COI, estos fueron enviados a la compañía Biotecnológica Macrogen (https://dna.macrogen.com/) para el procedimiento de secuenciación ADN por la tecnología Sanger doble cadena. Posteriormente, las secuencias fueron entregadas en formato FASTA (formato fichero informático basado en texto) para su respectivo análisis de caracterización molecular y filogenia.

### Caracterización molecular y filogenia

Las secuencias de ADN del gen COI generadas fueron liberadas en formato FASTA y analizadas en el programa bioinformático Análisis de Genética Evolutiva Molecular versión 11 (MEGA 11, por sus siglas en inglés https://mega.io/es) donde se originaron secuencias consenso a partir de las lecturas directa e inversa de cada muestra. Luego, fueron insertadas en la herramienta básica de búsqueda de alineación local (BLAST, por sus siglas en inglés https://blast.ncbi.nlm.nih.gov/Blast.cgi), donde se buscó similitud con secuencias de Ecuador disponibles en el portal del Genbank.

Para la caracterización molecular, las secuencias encontradas con similitud se alinearon con las de este estudio, luego se ingresaron y procesaron en el programa DnaSP v.5. Al tratarse de secuencias de un organismo diploide, se seleccionó la opción «Open Unphase/ Genotype Data file» para su importación. Los polimorfismos, su ubicación y los haplotipos a los que dieron lugar, fueron inmediatamente obtenidos mediante la opción «Generate Haplotype Data File» en un archivo de texto. 

Finalmente, para el análisis evolutivo, se construyó un árbol filogenético en MEGA11 empleando el método de agrupación unión de vecinos (*neighbor joining*, en inglés) ^26^, bajo el modelo de 3 parámetros de Tamura ^27^ con 500 réplicas de arranque, además de una secuencia de *P. rufotuberculatus* como grupo externo (MZ643673).

### Análisis de datos

Los haplotipos fueron organizados en hojas de cálculo del Software Microsoft Excel Versión 2021, indicando los polimorfismos comprendidos en cada uno de ellos.

### Consideraciones éticas

En el presente estudio, se emplearon ejemplares vivos de *P. chinai* provenientes del CICE, por lo que no fue necesario solicitar permisos específicos para su análisis. No obstante, para el uso de información genética correspondiente al gen COI de *P. chinai* de Ecuador, la cual se encuentra disponible en el banco de datos genéticos del NCBI, se incluyó el código de acceso correspondiente (MN504941 y MN504934), se citaron y referenciaron los autores de dicha información.

## RESULTADOS

### Electroforesis de productos PCR

Producto de la PCR dirigida a la amplificación del gen COI de los ejemplares *P. chinai* analizados, se obtuvieron amplicones con peso molecular de 710 pares de bases (figura 1).

**Figura 1:** Electroforesis en gel de agarosa al 2% de productos PCR diri-gida al gen COI. MPM: Marcador de peso molecular 1KB. CN: Control negativo. M1: *Panstrongylus chinai* de Jibito. M2: *Panstrongylus chinai* de Tambogrande. M3: *Panstrongylus chinai* de Las Lomas.

pb: pares de bases

### Caracterización molecular

Como resultado del secuenciamiento, se obtuvieron secuencias del gen COI de 650 pares de bases (pb), las cuales fueron alineadas para generar consensos del mismo tamaño. Tras la eliminación de 4 sitios con huecos, las secuencias se redujeron a un consenso final de 646 pb. Las secuencias consenso obtenidas se compararon con las depositadas en el banco de datos genéticos para Ecuador (códigos de acceso MN504941 y MN504934), observándose una alta similitud en una región de 393 pb, que fue utilizada para la caracterización molecular de *P. chinai*.

De las regiones de ADN de 393 pb analizadas, se excluyó un sitio con hueco, dando como resultado una región final de 392 pb. En este segmento, se identificaron 387 sitios monomórficos y 5 sitios polimórficos localizados en las posiciones genéticas 12, 189, 195, 226 y 252. Se observaron 6 mutaciones, correspondientes a los nucleótidos resaltados en amarillo (G, A, T), lo que dio lugar a la formación de 4 haplotipos: Hap-1, Hap-2, Hap-3 y Hap-4. Estos haplotipos se distribuyeron en 3 grupos, uno por cada localidad del norte de Perú, mientras que para Ecuador se identificó un único haplotipo (tabla 1).

**Tabla 1:** Características moleculares de las secuencias ADN del gen COI analizadas.

Especie	Origen geográfico		Fuente de secuencia	Haplotipos	Posición genética				
	País	Código			12	189	195	226	252
*P. chinai*	Ecuador	MN504941	Genbank	Hap-1	A	T	G	C	C
*P. chinai*	Ecuador	MN504934	Genbank		A	T	G	C	C
*P. chinai*	Perú	M1	Propia	Hap-2	A	T	A	C	C
*P. chinai*	Perú	M2	Propia	Hap-3	G	A	A	T	A
*P. chinai*	Perú	M3	Propia	Hap-4	A	T	A	C	T

### Análisis filogenético

El árbol filogenético, generado mediante el método Neighbor Joining, permitió inferir las relaciones evolutivas entre los haplotipos identificados en la caracterización molecular. Este análisis sostiene una hipótesis evolutiva en la que *P. chinai* de Ecuador forma un clado monofilético en relación con los ejemplares provenientes del norte de Perú. El haplotipo de *P. chinai* de Ecuador (Hap-1) resulta ser más reciente que los haplotipos observados en Perú (Hap-2, Hap-3 y Hap-4), lo que sugiere que la variante ecuatoriana podría haber surgido a partir de poblaciones peruanas de *P. chinai* (figura 2).

**Figura 2:** Árbol filogenético basado en el método de distancia *Neighbor Joining*^24^ y el modelo 3 parámetros de Tamura ^25^ con un valor *bootstrap* de 500 réplicas, que muestra la relación existente entre los haplotipos del gen mitocondrial COI hallados en las secuencias ADN de los especímenes del norte del Perú del presente estudio y las secuencias ADN de Ecuador obtenidas del *Genbank*.

En el árbol filogenético se observan cuatro clados: uno exclusivo para Ecuador (I) y tres correspondientes a Perú (II, III, IV). Los clados I, II y III se localizan en ramas subsecuentes que emergen de una rama compartida con el clado IV, lo que indica que todos provienen de un mismo nodo, sugiriendo un ancestro común. Este patrón se interpreta como un evento de cladogénesis, donde un linaje ancestral ha dado origen a linajes hermanos.

## DISCUSIÓN

Las especies de triatominos presentan un elevado grado de plasticidad morfológica y variabilidad en su distribución geográfica ^28^. Por esta razón, los entomólogos han estado llevando a cabo un análisis exhaustivo de diversos grupos de triatominos que exhiben diferencias morfológicas, las cuales a menudo son sutiles ^29^. Esto subraya la importancia de efectuar estudios moleculares en estos insectos, ya que es posible esclarecer de manera inequívoca la existencia de eventos evolutivos, como la especiación entre poblaciones de una misma especie.

En este estudio, se amplificaron fragmentos de ADN del gen COI de 710 pb a partir de ejemplares de *P. chinai* provenientes de Perú, de los cuales se analizó una región de 392 pb para la caracterización molecular. A partir de este análisis, se identificaron 4 haplotipos (Hap-1, Hap-2, Hap-3 y Hap-4), distribuidos en 1 haplotipo para Ecuador y 3 haplotipos para Perú, lo que evidencia una notable variabilidad genética entre las poblaciones de esta especie de triatomino. Este hallazgo es consistente con estudios previos que han documentado variaciones genéticas en poblaciones geográficamente distantes de *Triatoma infestans*, en los que se observó divergencia a nivel de especie ^30^, así como en otras especies del género *Triatoma*^31^. Estas variaciones sugieren que la geografía desempeña un papel fundamental en la diferenciación genética de las poblaciones. De manera similar, en estudios realizados con *P. herreri*, se han encontrado diferencias moleculares dentro de una misma población, lo que se interpretó como una posible especiación en proceso ^32^. Este fenómeno podría explicar la divergencia genética observada en *P. chinai* en el presente estudio, ya que las poblaciones de Perú y Ecuador están geográficamente separadas, lo que podría haber favorecido la acumulación de mutaciones independientes en cada población. Así mismo, la tasa de mutación del gen *COI* por sitio y por año ^21^ facilitó la identificación de polimorfismos que pueden ser indicativos de procesos evolutivos y de divergencia entre poblaciones. El marcador mitocondrial COI ha sido ampliamente utilizado en estudios de biodiversidad y filogeografía, especialmente en organismos invertebrados como insectos ^33^. En estudios previos sobre el género *Panstrongylus* se ha empleado el marcador mitocondrial *COI* debido a su alta tasa de mutación y capacidad para reflejar variaciones genéticas dentro de poblaciones, lo que permite inferir diferencias resultantes de procesos evolutivos locales o del aislamiento geográfico ^34^. Sin embargo, en las últimas décadas, el uso de otros marcadores moleculares como el *ITS-2* del ADN ribosomal también se ha utilizado en este tipo de estudios, ya que ha demostrado ser eficaz para diferenciar entre poblaciones y especies de triatominos ^35^^,^^36^. De manera similar, Jerí y Solis ^37^ emplearon este marcador para la diferenciación genética de especies de triatominos en zonas geográficas distintas.

Por tanto, desde un punto de vista teórico, la existencia de polimorfismos genéticos entre poblaciones de una misma especie se debe a la acumulación de mutaciones en los genomas a lo largo de sucesivas generaciones dando como resultado la formación de haplotipos distintos ^38^, algo que puede ser observado en marcadores moleculares distintos como el COI y el *ITS-2*, lo cual refuerza la existencia de variaciones genéticas entre triatominos separados geográficamente. Por ello, en nuestro estudio, las diferencias en los haplotipos encontrados entre las poblaciones de *P. chinai* de Perú y Ecuador pueden ser explicadas por este mecanismo, que refleja una diferenciación genética impulsada por la separación geográfica y la acumulación de mutaciones a lo largo del tiempo.

Los resultados obtenidos en el análisis filogenético basado en las secuencias del gen COI sugieren un proceso evolutivo de cladogénesis en *P. chinai* que apunta a un origen descendente de las poblaciones de Ecuador a partir de poblaciones del departamento de Piura, ubicado en el norte de Perú. Este patrón de relaciones filogenéticas es consistente con la proximidad geográfica y ecológica de ambas regiones. Las poblaciones de *P. chinai* en las provincias de Loja y El Oro, situadas en la región andina sur de Ecuador, están colindantes con la región norte del Perú ^39^, lo que proporciona un contexto biogeográfico favorable para el intercambio de individuos y material genético entre las poblaciones de ambos países. La cercanía geográfica, junto con el hecho de que las secuencias de ADN de Ecuador analizadas en el presente estudio provienen de ejemplares capturados en 2019 en áreas cercanas a la frontera con Perú ^24^, refuerza la hipótesis de un vínculo histórico y continuo entre estas poblaciones. El desplazamiento de *P. chinai* desde Perú hacia Ecuador y su relación en términos de distribución geográfica no parece ser un fenómeno reciente. De hecho, un estudio exhaustivo realizado en 2010 en el sur de Ecuador, específicamente en zonas rurales de la provincia de Loja, documentó la presencia de *P. chinai* tanto en ambientes intradomiciliarios como en áreas peridomiciliarias. Además, en dicha región se encontraron otras especies de triatominos, como *R. ecuadoriensis*, *T. carrioni* y *P. rufotuberculatus*^40^. Los informes sobre las especies del género *Panstrongylus* en el Perú ^41^ indican que *P. chinai* es una de las especies más prevalentes en las regiones norte y nororiental del país ^42^, lo que resalta su importancia como vector de *Trypanosoma cruzi*, el agente etiológico de la enfermedad de Chagas. La predominancia de esta especie en áreas específicas de Perú es relevante para la comprensión de los patrones de transmisión del parásito y las dinámicas de la enfermedad. En este sentido, la distribución de *P. chinai* en el país ha sido documentada en varios estudios, que señalan su presencia desde al menos 1961 ^43^, con nuevos informes en 1986 ^44^, lo que sugiere una presencia estable y prolongada en estas áreas geográficas. Por tanto, los resultados obtenidos en este estudio pueden ser dilucidados, ya que muestran una conexión filogenética entre las poblaciones de *P. chinai* de Perú y Ecuador, la cual se basa en la existencia de un flujo e intercambio genético continuo entre las poblaciones de ambos países. Dado que las poblaciones de *P. chinai* en las regiones fronterizas de Perú y Ecuador comparten características ecológicas similares, facilitando la propagación de la especie y, potencialmente, del parásito *T. cruzi*. Además, la proximidad geográfica entre las regiones endémicas de ambos países favorece la interacción constante entre las poblaciones de triatominos, lo que aumenta la probabilidad de que este flujo continúe.

Las implicancias en salud pública derivadas de nuestro estudio están estrechamente relacionadas con la distribución espacial de *P. chinai* en el norte de Perú y Ecuador, ya que ambos países se encuentran en una región de América del Sur en la que, desde hace varias décadas, se reportan casos de la enfermedad de Chagas. Este hallazgo cobra relevancia en el contexto de la creciente urbanización, la migración de comunidades hacia zonas rurales y los efectos del cambio climático, factores que están alterando los patrones epidemiológicos de la enfermedad. De acuerdo con la Organización Mundial de la Salud (OMS), el cambio climático y los movimientos migratorios han transformado el panorama epidemiológico de la enfermedad de Chagas, contribuyendo a su expansión y convirtiéndola en una condición de alcance global en un tiempo relativamente corto ^45^. En el caso de Perú, para el año 2023 se reportaron 57 casos confirmados y 7 probables de enfermedad de Chagas ^46^, con una actualización para la semana epidemiológica 11 del año 2024, que reflejó 3 casos confirmados y 9 probables ^47^. En Ecuador, durante el año 2023, se reportaron 118 casos confirmados ^3^. Estos datos subrayan la importancia de continuar con la vigilancia epidemiológica y la implementación de estrategias de control, especialmente en áreas geográficas donde la distribución de *P. chinai* podría contribuir al mantenimiento de ciclos de transmisión.

Nuestro estudio presentó algunas limitaciones que deben ser consideradas para enriquecer y mejorar la interpretación de los resultados obtenidos. Una de las principales limitaciones fue la escasa información bibliográfica disponible sobre el tema de estudio, lo que dificulta una comparación más exhaustiva de los hallazgos en el contexto de investigaciones previas. Además, la cantidad de datos genéticos sobre el gen COI de *P. chinai* en el banco de genes es limitada. En este sentido, solamente se encontraron secuencias depositadas por Barnabé *et al*. ^24^, lo que restringe la posibilidad de efectuar un análisis comparativo más amplio con otras poblaciones o especies relacionadas. De hecho, el presente estudio representa uno de los pocos trabajos en los que se ha analizado el gen COI en *P. chinai*, lo que subraya la importancia de generar y compartir más datos genéticos sobre esta especie para futuras investigaciones.

En conclusión, nuestro estudio proporciona una visión general sobre la distribución espacial de *P. chinai* entre el norte de Perú y Ecuador, analizando sus variaciones genéticas. Se identificaron características moleculares que distinguen a las poblaciones en términos de polimorfismos, aunque filogenéticamente se encuentran emparentadas en términos de cladogénesis y ascendencia común. En este sentido, se plantea una hipótesis evolutiva en la que las poblaciones de *P. chinai* del norte de Perú habrían dado origen a las poblaciones presentes en Ecuador. Estos resultados señalan la importancia de llevar a cabo estudios moleculares adicionales sobre la distribución de triatominos en América del Sur, ya que la información obtenida contribuirá al diseño de estrategias preventivas para el control vectorial, especialmente en las zonas limítrofes entre países endémicos de la enfermedad de Chagas.
